# Implementation of Assistive Technologies and Robotics in Long-Term Care Facilities: A Three-Stage Assessment Based on Acceptance, Ethics, and Emotions

**DOI:** 10.3389/fpsyg.2021.694297

**Published:** 2021-08-26

**Authors:** Annette Franke, Elmar Nass, Anna-Kathleen Piereth, Annabel Zettl, Christian Heidl

**Affiliations:** ^1^Ludwigsburg Protestant University of Applied Sciences, Ludwigsburg, Germany; ^2^Cologne University of Catholic Theology, Cologne, Germany; ^3^SRH University of Applied Sciences, Fuerth, Germany; ^4^YOUSE GmbH, Berlin, Germany

**Keywords:** assistive technologies, assistive robotics, care homes, long-term care, ethics, organization, emotion management, quality of life

## Abstract

Assistive technologies including assistive robots (AT/AR) appear to be a promising response to the increasing prevalence of older adults in need of care. An increasing number of long-term care facilities (LTCFs) try to implement AT/AR in order to create a stimulating environment for aging well and to reduce workload for professional care staff. The implementation of new technologies in an organization may lead to noticeable cultural changes in terms of social interactions and care practices associated with positive or negative emotions for the employees. This applies especially for LTCFs with high rates of vulnerable residents affected by increasing care needs and specific ethics in nursing and cultural rules within the setting. Thus, systematic consideration in leadership management of emotions and ethical aspects is essential for stakeholders involved in the implementation process. In this article, we explicitly focus on the emotions of the employees and leaders within LTCFs. We relate to direct consequences for the organizational well-being and culture, which is of course (indirectly) affecting patients and residents. While aspects of technology acceptance such as safety and usefulness are frequently discussed in academic literature, the topic of emotion-management and ethical questions during the organizational implementation process in LTCFs received little attention. Emotional culture entails affective values, ethical norms and perceptions of employees and further investigation is needed to address the importance of transformational leadership during implementation process. For this purpose, we developed a three-staged assessment tool for implementation of AT/AR in long-term care institutions. Acceptance (A), ethical acceptability (A) and emotional consequences (E) are considered as comprehensive assessment, in which emotional consequences comprise management aspects of transformational leadership (T), emotion-management (E) and organizational culture (O). Based on AAE and TEO, this paper presents an integrated framework illustrated with a illustrative example and aims to combine established approaches with ethical insights in order to unfold potentials of AT/AR in LTCSs.

## Introduction

Digitalization 4.0 and assistive technology including assistive robotics (AT/AR) evoke certain aspirations in health care with regard to well-being and economic arguments (Magsamen-Conrad and Checton, 2014). In March 2018, the World Health Organization (WHO) promoted the access to AT/AR for all member states:

*“The impact of assistive technology goes far beyond the benefits of health and well-being to individual users and their families. It also has socioeconomic benefits, by reducing direct health and welfare costs (such as hospital admissions or state benefits), enabling a more productive labor force, and stimulating economic growth” [World Health Organization (WHO), 2018, p. 3].*

AT/AR embrace various dimensions of interesting products, aids, devices or software which enable persons with functional losses, e.g., communication boards, screen readers, positioning devices or service robots^1^ [International Federation of Robotics (IFR), 2021]. In 2019, the market for professional service robots increased by 32% [International Federation of Robotics (IFR), 2020]. Growing potential can be assumed, when more robots could overcome the prototype stage (Bedarf et al., 2015; Hersh, 2015; Scassellati and Vázquez, 2020).

Within the last decades information, communication and assistive technologies seem to find their unstoppable way into (health-)care facilities, even when many institutions in residential care—compared to technological pioneers in long-term care like Japan—are still struggling with general preconditions for new technologies (e.g., access to wireless internet) (Moyle et al., 2018; Seifert et al., 2020; Grüneberg, 2021; Tan et al., 2021).

Especially for decision makers in long-term care facilities (LTCFs)^2^ AT/AR suggests new opportunities related to preserving activities and personal well-being for residents, lower health care costs, reduced workload for professional caregivers and solutions for nursing shortage (Sharkey and Sharkey, 2012; Bonaccorsi et al., 2016; Scassellati and Vázquez, 2020; Tan et al., 2021).

Robots are supposed to be used in long-term care for different purposes: to solve logistical challenges, as supportive aid for basic needs, for safeguarding and monitoring, and for instrumental tasks like cleaning or social interaction (Tan et al., 2021). At the same time, LTCFs represent particular challenging settings including self-image of care and specific vulnerable clients. In Germany for example, about a quarter of all persons in need of care are living in LTCFs, of whom more than 70% suffer from severe impairment of independence (Destatis, 2020).

In this respect, AT/AR may lead to fundamental organizational changes in LTCFs and the question is not if or when, but “how best to shape and direct our efforts to optimize the development and application of new technologies” (Schulz et al., 2015, p. 725).

AT/AR already posed a controversial discussion about safety, social justice, usefulness and appropriateness in long-term care. In addition, discussions arose on questions of economic viability, customer acceptance, co-design and user impact (e.g., stress level) (Berkowsky et al., 2017; Chu et al., 2017; Kwon, 2017; Meyer and Fricke, 2017; Diefenbach, 2018; Merkel and Kucharski, 2019; Peine and Neven, 2020). All these aspects have already been explored in current research literature, even though a deeper understanding, for example, of ambivalent user acceptance is still needed (Hersh, 2015). In this respect, Krick et al. (2019), provide a systematic review on three core outcome dimensions concerning digital technologies for all participants involved in the care sectors by focusing on *acceptance, effectiveness and efficiency* (AEE) of new technologies.

While these dimensions of AT/AR in long-term care are frequently discussed in academic literature, *emotional and ethical* acceptance and acceptability, and the *role of leadership* within organizations received little attention so far. Even though some studies underline the importance of implementation circumstances and management practices (e.g., further education of employees) (Tan et al., 2021), deeper investigations are required in terms of management approaches as Melkas et al. (2020, p. 5) point out: *“*Robotic research has so far focused on technical implementation, technology development, and clinical applications, but there has been limited discussion on social and managerial issues that might be equally important for successful robot use.*”*

The aim of this article is to address stakeholders and management staff in LTCFs in their strategic leadership role in the AT/AR implementation process. From our perspective, it is important for this target group to examine “emotion-management,” which means the recognition of ethics and emotions (explicitly from staff and implicitly from LTC-residents) within the organization (Thiel et al., 2012). As the majority of LTC-residents experience multimorbidity, cognitive impairments, functional decline and decreasing quality of life (e.g., lower levels of loneliness) (Boggatz, 2020), LTCFs represent a specific workplace environment, where the demanding conditions of residents and social norms frame multiple dimensions of emotionality (Bolton, 2001). Fulfillment of professional care tasks like conversations, lifting or washing by different kinds of robots, has emotional and ethical consequences for both patients and staff in care homes. This applies in particular in consideration of nursing values like autonomy, beneficence, justice and non-maleficence (Beauchamp and Childress, 2001; Sharkey and Sharkey, 2012; [Bibr B31][Bibr B75]). In addition, anxiety and skeptical expressions of nursing staff can be assumed in the fear to become replaced by technology (Feil-Seifer et al., 2007; Broekens et al., 2009; Wolbring and Yumakulov, 2014; Coco et al., 2018; Mitzner et al., 2018). From a practical perspective, this means that strategic planning and understanding of significant affects and emotions are critical to the ongoing success of a modern organizations (Thiel et al., 2012).

We therefore combine Bolton (2005) idea of “*emotion-management*” and the concept of “*transformational leadership*” according to Bass and Avolio (1994). Bolton developed a multidimensional approach to emotionality in organizations with specific focus on nurses, in critical engagement with Hochschild (1989) distinction on emotional work and emotional labor. For Bolton, feelings and motivations are central in interactions within an organization, where actors (leaders as well as workers) are able to channel emotions in their workplace to achieve organizational goals. Workers are purposive agents of their emotions, but constrained by organizational emotion rules and embedded in broader cultural beliefs and values. Bolton provides a typology of four dimensions of emotion-management, from which we depict “prescriptive emotion-management” as most relevant for our topic (= emotion-management according to organization/professional rules). This means, that organizational power is related to emotional rules, as Bolton (2005, p. 8) labels “nurses as multi-skilled emotion managers” (see also Cranford and Miller, 2013). The approach of transformational leadership we refer to, underlines the role model function of leaders, setting attractive goals and fostering motivation and stimulation for employees (Bass and Avolio, 1994). Leaders and management staff express themselves through aspects such as inspiration, vision or personal role model action and thus deliberately appeal to emotions of their employees.

Drawing on both concepts, two questions arise as relevant for this paper: (1) How can emotion-management and transformational leadership fruitfully contribute to AT/AR implementation within LTCFs? (2) How can a holistic assessment of emotional and ethical aspects be designed as part of the change management process for AT/AR implementation in LTCFs without neglecting the acceptance-perspective?

To address these questions, we apply the AEE system by Krick et al. (2019) as an assessment tool and develop it further with regard to ethics and emotionality in organizations. First, we present an ideal-typical, but concrete illustrative example of such technology, which we see as the central thread of our presentation (2). After that we outline general practical consequences of AT/AR implementation in the care sector for organizational culture in view of our illustrative example (3). In the following section, we systematically illustrate our assessment criteria with regard to emotions and ethics (4, 5), and apply it to the illustrative example in three stages (6). We conclude with a look at our contribution to the discussion, followed by an outlook (7).

## Illustrative Example

**FIGURE 1:**
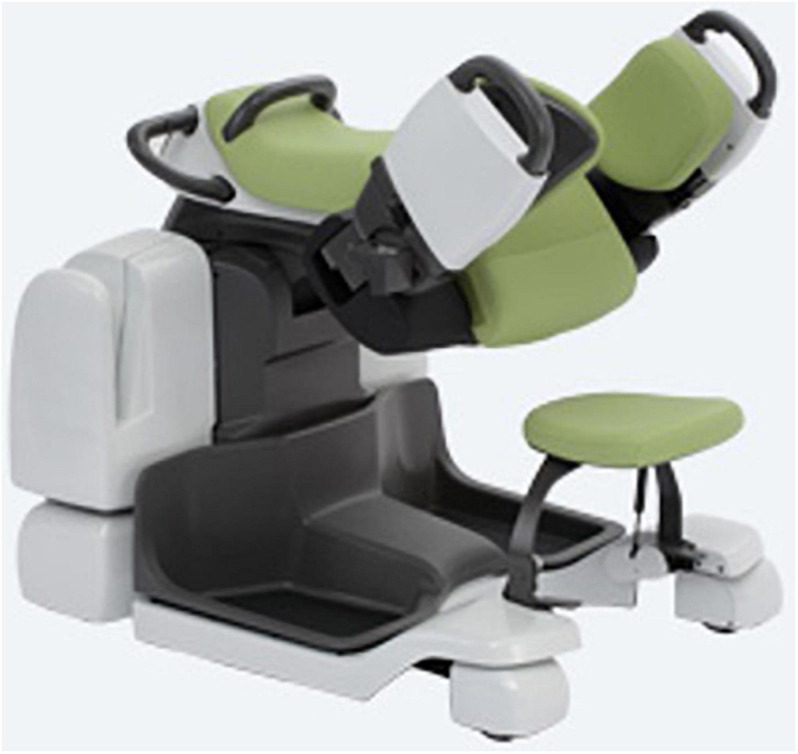
Care Assist Robot (Source: Future of Occupation, 2015).

**FIGURE 2:**
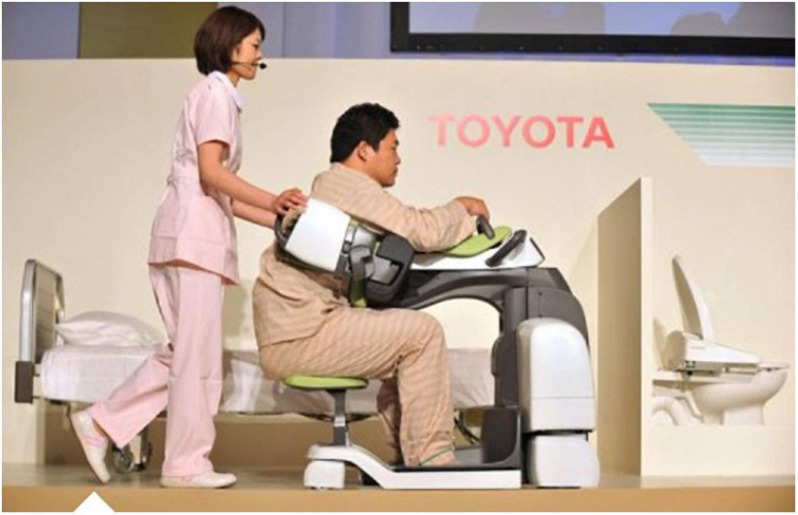
Care Assist Robot (Source: Future of Occupation, 2015).

Some hurdles and opportunities of negative emotions and concerns of acceptance and acceptability are typical challenges for AT/AR implementation processes (Diefenbach, 2018). An inpatient LTCFs is planning on implementing the “Care Assist Robot” (CAR, Figures 1, 2; Toyota, 2021). The facility has 61 bed spaces, of which 21 patients are cognitively impaired and in need of severe care, and predominantly require 24-h assistance. CAR is to be used for people with multimorbid diseases as well as physical limitations during early and late duty. As Figures 1, 2 show, it will be used in work processes such as transfers and lifting, e.g., from bed to wheelchair, or in personal hygiene processes from the wheelchair to the bathtub. CAR is thereby operated by a caregiver. Nursing staff, nursing assistants and care assistants are to be supported and relieved (and, among other things, skeletal and muscular disorders on the part of the nursing staff are to be reduced preventively), because lifting and transfer services are physically very strenuous (Future of Occupation, 2015).

The organization also expects a significant reduction in costs and compensation for the existing shortage of nursing staff through the use of CAR. Furthermore, the implementation is supposed to increase the quality of work and the job satisfaction of the employees. The robot is expected to be implemented within the next months.

Employees in management positions (nursing service management and residential area management) were included in the selection process as well as in the discussion with the company, which manufactured CAR. In doing so, they were able to consider the suitability in terms of content and function on the basis of a catalog of criteria as well as the price-performance ratio. The acquisition costs for three CAR amount to € 28,600.

The implementation process, which is going to take about 3 months, will be supported by the manufacturer, who will hold a workshop every 6 weeks to inform employees about the use of the robot, how to handle it, how to document data, and how to change processes and workflows. Four weeks after the implementation the manufacturer will hold an additional workshop.

Several employees report anxiety, anger, bewilderment and disappointment. While some of them are frustrated and will not accept that management is buying robots instead of creating new jobs, others are concerned about being replaced by a robot, with potential job loss. In addition, some nurses are afraid of their patients with dementia, who could possibly be confused by CAR and perceive additional stress caused by technology-generated overload for sensory and motoric functions (hearing, collision with CAR in the room).

For some nurses CAR appears like a dystopian scenery with hybrid and blurring boundaries between humans and machines (“Who is taking care of a person? The robot or the person, who is pushing the button? And what comes next?”). Even when some persons are also curious and excited to try CAR, they hesitate whether they can operate the machine.

The consequence, or possible unexpected reactions of staff, could be that more negative emotions arise within the organization and this could result in, for example, a disturbed relationship of trust with the home’s management and leaders, increased illness-related absence or even increased staff turnover. On the other hand, however, some other colleagues report a rather positive attitude toward robots and are looking forward to possibly having more joy and meaning in performing their tasks again.

With our example, we tried to point out the possible dynamics of emotionality, which are important for our further argumentation. For the next step, we have to focus on organization consequences of AT/AR implementation in care homes to get a broader picture and better understanding of the upcoming assessment part.

## Consequences of AT/AR Implementation for Organizational Culture in the Care Sector

In practical implementations, it has so far mostly been assumed that AT/AR “only needs to be activated” in order to have a positive effect (Beimborn et al., 2016; Gallistl and Wanka, 2019; Merkel and Kucharski, 2019). However, many hurdles with regard to individual characteristics, participatory design or contextual factors can be observed in connection with the implementation of digital technologies in professional everyday (health-)care:

Granja et al. (2018) examined in their systematic review major facilitators and barriers of eHealth-implementation in a total of 221 included studies. On the one side, *“Quality of healthcare”* turned out to be the most relevant category contributing to the success of eHealth interventions in clinical practice. This embraces *inter alia* better communication with the patient, improved diagnosis and provided patient-centered care. The determinant *“workflow”*—i.e., the manner people interact with their work, other people and communication pathways—plays a critical role in affecting the adoption at this point. It is imperative to mold the new work processes after the intervention in a way that increased workload, workflow disruption, undefined roles, undermined face-to-face communication as well as staff turnover are prevented (Granja et al., 2018). However, *“costs”* was the category most mentioned adding to the failure of eHealth interventions due to the fact that the shortage of financial resources inhibits the AT adoption or rather implementation. It becomes clear that a national policy for investment in eCare-Technologies—especially establishing financial mechanisms to support organizational changes—is required for successful product launches (Granja et al., 2018, p. 4–5).

Kruse et al. (2018) analyzed 30 articles and identified 33 different barriers of which the most occurred and important ran as follows: “*technically challenged staff*,” “*resistance to change*,” “*cost*,” “*reimbursement*,” “*age of patient*,” as well as “*level of education of patient.*” Against this backdrop, the reviewers come to conclusion that individual training and organizational change-management techniques are essential to overcome the outweighing technology-specific barriers (Kruse et al., 2018, p. 4).

Papadopolous et al. (2020) explored implementation-enablers and -barriers in a systematic review for the field of socially assistive humanoid robots in health and social care. The 12 in the analysis included studies suggest that *“personalization”* and *“enjoyment”* seem to be crucial adoption-enablers. In contrast, *“technical problems”* and the *“limited capabilities of the robots”* were summarized as the two most important obstacles. It should be noted that the evidence of investigated studies was limited, whereby a generalization of these initial findings is excluded (ibid.).

Melkas et al. (2020) identified six types of impacts on care personnel (five types of impacts on care residents) concerning the use of “Zora,” a care robot for personal cognitive and social assistance. For employees changes have been experienced in terms of “*work atmosphere,”* “*meaningfulness,”* “*workload,”* “*professional development,”* “*competences,”* and “*work ethics”* and the authors underline the importance of organizational leadership and information policy within the organization (ibid.).

The study of Mitzner et al. (2018) also focused on the perception of professional caregivers with a mobile manipulator robotic assistant. The participants reported some positive experiences of AR, e.g., *“time efficacy”* and *“productivity,”* but at the same time skepticism in terms of *“reliability,” “appropriate tasks”* for individual difficulties, and the question if the robot *“might be a hazard”* for some patients.

Attitudes of healthcare staff which come into conflict with the AT and AR-adoption potentially result in skepticism, frustration, negative thoughts and bad feelings (Magsamen-Conrad and Checton, 2014; Melkas et al., 2020). Slightly or strongly negative emotions of patients and healthcare professionals appear as underestimated barriers resulting in the AT/AR never completely integrated into the workflow, whereby the implementation fails overall (Nielsen and Mathiassen, 2013; Sølling et al., 2014).

As a way of summarizing, elected key implementation dimensions being based on the illustrated articles are synoptically organized by the perspectives of patients, healthcare professionals as well as home and nursing management staff (Table 1).

**TABLE 1:** Elected key dimensions influencing the success or failure of AT-implementation by perspective (own elaboration).

	Perspective		
Outcome	Patients	Healthcare professionals	Home and nursing management staff
**Success**	Patient empowerment and self-management Enjoyment Personalization	Quality of healthcare Usability Familiarization	Costs Privacy Data and effectiveness policies Successful change management
**Failure**	Age Level of education (esp. AT literacy) Privacy and security	Technically-challenged staff Resistance to change Workflow Perception of impersonal care	Costs/reimbursement Privacy/confidentiality Data security Effectiveness

Notably, not merely the organizational environment influences the deployment (at best, in a positive way) but the implementation itself affects the institution. In this light, Kuziemsky et al. (2016) investigated in a systematic review the phenomenon of so called *“unintended consequences”* (UICs) as well as organizational and social issues related to these effects.

The term UICs was established as crucial factors, which can be beneficial and/or adverse with a lack of purposeful action of causation. Although the relationship between collaborative teams and individual providers were determined as the main source of UICs, there is a need to study and substantiate the reason and manner of their occurrence. Beside this, the UICs are contributing to diverse organizational and social sub-themes, namely: process change and evolution, individual-collaborative interchange, context of use as well as (proactive) approaches to model, study, and understand UICs (Kuziemsky et al., 2016).

A particular highlight was the realization that UICs go beyond errors and also include changes in workflow, communication and emotions (Ash et al., 2007; Borycki et al., 2012). Moreover, UICs can beneficially elicit positive processes and thus improve care delivery (Ash et al., 2007; Melby and Hellesø, 2014). For this purpose, UICs should be better explored to anticipate the consequences and then to specifically, proactively use them in the phase of pre-implementation on the micro and meso level of a care institution (Kuziemsky, 2015).

In summary, it can be emphasized that the nursing staff in management positions should constantly be aware of the potential development of UICs during AT/AR implementation. On that account it is important to know which enablers or obstacles are decisive for an accomplished deployment. In this way in the case of suboptimal or faltering implementation progresses high priority categories (i.e., the category “workflow”) can be particularly analyzed and influenced by organizational and emotional aspects.

## Organizations as Emotional Arenas for Transformative Leadership

One of the most prominent perceptions of emotionality and management leads to a distinct divide between private and public spheres, where emotions may occur. For example, Hochschild (1989) distinguishes between unpaid “emotion work” and paid “emotional labor.” She argues, that emotion work can be defined as a person’s management of her or his internal feelings, with the aim to evoke a specific emotional reaction from another person (private context). Translated into a professional context, for example when leaders ask their employees to do “emotion work” in contact with their clients, this “emotion work” turns into “emotional labor.” In general, Hochschild’s concept refers to service activities or activities related to people. Emotional labor for example entails frequent telephone or personal contact with clients or customers and requires a certain emotional expression toward them. However, if feelings are suppressed systematically and for a longer period of time or employee and client indicate different feelings and unequal exchange of emotions, this leads to negative effects on mental health of the employees and impaired organizational well-being.

Fineman (1993) places emotions explicitly in the workplace. He denotes organizations as “emotional arenas.” Everyday dissatisfactions and disillusions, as well as devotion and passion, such as boredom, envy, fear, love, anger, guilt, infatuation, etc., bring about the potential to unite, but also to separate the workforce. They determine, how roles are appropriated and implemented, how positions of power are exercised, trust is lived, acceptance and commitment is developed and how judgments are made in a way, that they cannot simply be excluded from organizational processes (ibid.).

With her concept of “emotion-management” primarily based on gynecologic nursing practice, Bolton (2005) underlines the difficulties to separate private and public emotional dimensions in interactions between employees. In doing so, she criticizes Hochschild’s distinction of emotion work and emotional labor:

*“Emotion is a lived, interactional experience with traffic rules of interaction framing how it is expressed and shared. Employees draw on both professional, organizational and commercial codes of conduct and social feeling rules in their interactions with others. The fragile accomplishment of social interaction is continually maintained through, not only formal exchanges, but also through episodes of compassion and shared laughter”* (Bolton, 2005, p.2).

The approach assumes that emotions do not simply “happen” in organizations, but are the result of controlled processes in which employees are depicted as active agents. Accordingly, managing emotions does not mean imposing unauthentically emotions, but rather creating the possibility for emotional compliance. Bolton’s model of “emotion-management” is based on four categories:

• *“presentational”* (emotions are handled according to social rules),
• *“philanthropic”* (emotion-management as a “donation”),
• *“prescriptive”* (with regard to organization or professional rules),
• *“pecuniary”* (emotion-management is commercialized).

Drawing on employees in LTCFs, emotion-management is framed by specific social norms on acceptance and acceptability of AT/AR. “*[I]n this way nurses are portrayed as multi-skilled emotion managers who both comply with and resist the organizational constraints which exist around them*.” (Bolton, 2005, p.8). Aside organizational rules, Cranford and Miller (2013) underline the role of organizational “signals” as important for persons in need of care.

For example, Smollan and Sayers (2009) focus on the affective organizational culture, which shapes how emotions are experienced and expressed. The results of their study show an interconnection of cultural change and emotions: the greater the congruence between the values of the employees and the organization, the more positive are reactions of the employees with regard to change. If the emotions of employees are treated with respect and appreciation during an organizational culture change process, employees will participate more in the change process (ibid.). Kaplan et al. (2014) developed a theoretical model which highlights eight specific categories of leader emotion-management behavior like interacting and communicating in an interpersonally tactful manner, demonstrating consideration and support for employees or structuring work tasks with consideration for employees’ emotions (ibid.). In addition, the authors address dimensions that include knowledge (e.g., self-awareness, knowledge of emotions and their consequences, etc.), skills (e.g., emotion recognition, perspective taking, etc.) as well as proximal outcomes (psychological safety, satisfaction with the leader, etc.), and ultimate outcomes (cohesion, satisfaction and organizational commitment).

Also the study from Höld et al. (2020) shows, that cooperation with the team and supervisors is one of the most significant aspects of job satisfaction among professional nurses. A good team can create professional and ideal support for nurses and improve their professional development and quality of care. These positive aspects of job satisfaction should be integrated in emotion-management and play a significant role in the implementation of AT/AR. In this respect, leaders are requested, who are able to perceive emotions in a targeted manner within the framework of their management behavior, who are able to show these emotions and to evoke them in employees. A mutual exchange or implementation of emotions within emotion-management for prospective change management processes is therefore desirable.

If we now focus on leadership within the organization and specifically on theories of leadership, it becomes apparent that emotions also play a significant role here (Kaplan et al., 2014; Schein and Schein, 2018). Stakeholders and management staff have to shape the implementation process in a way that it matches the assumptions and values of the employees, their organizational culture, to ensure their acceptance. Emotions are interwoven in leadership theories and are at the heart of many leadership mechanisms, such as inspired employees, sustainable interpersonal relationships and investment in employee development, performance and satisfaction, etc. (MacGregor Burns, 2007; Little et al., 2016). As a result, a large number of academics understand persons in leadership roles as managers of group emotions (Brotheridge and Lee, 2008; Ashkanasy and Humphrey, 2011; Little et al., 2016).

“Transformational leadership” according to Bass and Avolio (1994) provides a model for emotion-management based on four principles:

(1) “*Idealized influence”* represents the role model function of leaders. A clear orientation of values, which is reflected in the attitude of the leader, provides orientation for employees. Furthermore, the authors emphasize that in this form of leadership, managers put their own interests behind those of the organization as a whole. Such leadership behavior triggers respect, admiration and trust among employees.
(2) “*Inspirational motivation”* is about motivating employees through challenging and attractive goals. The meaning of these goals must be made clear. By pursuing a common goal, team spirit, optimism and commitment can be fostered.
(3) The approach also emphasizes the role of “*intellectual stimulation”* of employees. Creativity and problem-solving skills of employees should be promoted. In the long term, employees should acquire the ability to critically question outdated assumptions, routines and habits and find new approaches to solutions.
(4) Within the framework of “*individualized consideration,”* employees should be individually supported according to their personal strengths, weaknesses and expectations. The leader acts as a kind of coach and promotes the development of the professional perspectives and potentials of the individual to a higher level (ibid.).

Transformational leaders express themselves through aspects such as inspiration, vision or personal role model action and thus deliberately appeal to emotions of their employees in order to support them as well as consecutively raise their acceptance, aspirations, motives and goals (Bolton, 2005). Transformational leadership behavior is intended to generate optimism, confidence and belief in their employees by suggesting to them that although their challenges seem immense (Bass, 1985; Yukl, 2012).

The implementation of new technologies in an emotional-sensitive setting like LTCFs require specific leader behavior in respect of the identity and stability of the care personnel. Following the approaches of emotion-management and transformational leadership, the successful implementation and realization of profound change and innovation processes can be decisively supported by shaping an emotional relationship between leaders and those led (Tichy and Ulrich, 1984). In light of the aforementioned concepts, an integrated acceptance model could be useful, which comprises key dimensions for assessment of AT/AR implementation in long-term care institutions.

## A Multi-Perspective Model of Acceptance, Ethical Acceptability and Emotional Consequences

Following the work of Krick et al. (2019), which used the three outcome dimensions acceptance, effectiveness and efficiency (AEE) in terms of AT/AR evaluation, we present a modified approach based on the three perspectives *acceptance (A)*, *ethical acceptability (A)* and *emotional consequence (E)*. Together, they form the acceptability and emotional consequences (AAE) model according to our perspective. In a subsequent step, we apply this model to our illustrative example and use it to arrive at a holistic evaluation of the implementation for the organizational culture.

### Acceptance (A)

When addressing the issue of technology acceptance, it is important to consider the needs and characteristics of potential users. Persons in LTCFs are more frequently confronted with experienced functional losses and decreased coordinative and sensory abilities. These difficulties cause individuals to perceive, use and accept technology differently.

One of the most prominent concepts is the “Technology Acceptance Model (TAM)” by Davis (1986). This model highlights the usefulness of a technology (degree to which a person believes that using a particular system would enhance her or his performance) and the perceived ease of use (degree to which a person believes that using a particular system would be free of effort) which together with external factors influence the attitude toward using and the behavioral intention to use (ibid., 320). Critical arguments, however, address the limited practical implications of this approach and the influence of professional or occupational use of a system (King and He, 2006).

The further developed TAM2-Model (Venkatesh and Davis, 2002) considers the social and cognitive instrumental factors influencing the perception of usefulness such as norms, image, job relevance and voluntariness. Thus, TAM2 underlines that both, social and cognitive-instrumental variables, have an impact on technology acceptance and use. In this context, the model is sufficient, when a person, even if he or she does not support a certain behavior, still engages in it if he or she assumes that someone personally important approves of it.

Venkatesh et al. (2003) developed the Unified Theory of Acceptance and Use of Technology (UTAUT) by integrating several other concepts such as TAM, theory of planned behavior (TPB) or theory of reasoned action (TRA). The concept underlines the importance of social influence and facilitating conditions in acceptance of technologies whereas variables as age, gender or experience only have a moderating effect. The most important variable for behavior and use of technology represents the own performance expectancy of a person. In the TAM3-Model, Venkatesh and Bala (2008) focused on the perceived ease of use, which is influenced by factors such as computer self-efficacy, computer anxiety or results demonstrability.

All TAM-models in their modification are considered to have been empirically tested many times. However, these models have hardly been applied in relation to persons in need of care or in care homes.

In the Almere model, Heerink et al. (2010) used the items of the former UTAUT questionnaire adaptively with regard to animal-like social robotics and older persons as their users. Instead of expected performances and expected effort, the authors renamed the variables with “perceived usefulness” and “perceived ease of use.” In addition to the existing assumptions that usefulness and voluntariness play a significant role in how a person accepts AR, Almere also emphasizes the importance of affective and cognitive attitude. Thus, acceptance variables have been added to the UTAUT model like perceived enjoyment, confidence, or perceived adaptability. In total, 12 different dimensions determine technology acceptance such as anxiety (for using social robotics), (positive or negative) attitude toward technology, facilitating conditions (adequate introduction in functions of the robot), intention to use, perceived adaptiveness (of the robot with regard to specific needs of the patient), perceived enjoyment, perceived ease of use, perceived sociability, perceived usefulness, social influence (related to the acceptance of others), social presence (as perceived social interaction with the robot) and trust (integrity and reliability of the robot). Central to the model is thus not only the individual perspective, but also the idea that acceptance is embedded in social contexts, like in our example in an organizational setting.

In summary, these prominent and empirical tested models for technology acceptance focused on various aspects of acceptance such as perceived usefulness, ease of use, or voluntary in use. In the prominent triangle of caregivers, care recipients and technology, organizational culture and ethics were, however, mentioned only in passing.

### Ethical Acceptability (A)

For an ethical evaluation acceptance alone is not a sufficient criterion. A humane perspective does not first ask about the usefulness of a technology for solving concrete practical problems or a mere acceptance. It asks about acceptability against the background of the consequences of a technology’s use for the image of humanity and coexistence. First of all, AT/AR in care contexts are undoubtedly something good, if what they help to achieve in turn produces something good in the end.

Justice, self-determination, privacy, etc., and the criteria for nursing formulated by Beauchamp and Childress (2001) like justice and autonomy are only a first approach here. Going to the root of the ethical question, self-determination is by no means the fulfillment of one’s own desires. With Immanuel Kant, autonomy means: reason, free from egoistic willing, recognized what a person should do. If a person makes this ought her/his ought, she/he is ultimately autonomous and free. But this has little to do with the common understanding of self-determination; for it is the obligated freedom in responsibility. Thus, there is a significant need to semantically fill the ethical generalities and to substantially question, evaluate and responsible implement the use of technology to avoid unintended consequences. The possible reference to the distinction that robots can only achieve predetermined goals but cannot set goals for themselves is no longer sufficient when algorithms inspire and control each other even without human intervention (Matsuzaki and Lindemann, 2016). Is the care robot seen as a colleague or is it even allowed to determine work processes of human employees? This, however, contradicts our notion of humanum and also implies highly complex liability issues in case of robot errors (Kaplan, 2004; Bartneck et al., 2019; Nass, 2020). Who will take responsibility for this?

We first take a look at already existing instruments of ethical evaluation of AT/AR before we sharpen and propose our position. An ethical evaluation-model popular in the context of Ambient-Assisted-Living (AAL) is the MEESTAR-Model (Model for the Evaluation of Socio-Technical Arrangements) (Weber, 2015, 2020). It takes an explicit ethical view on the consequences of AAL-adoptions and interventions like privacy, security or justice and tries to balance responsibility and ability in this context. These aspects are remarkably necessary with regard to clients with cognitive impairments in order to provide appropriate information and tools for a sensitive adoption of technology. In the course of further development, less “practical” approaches and more questions of attitude, participation, trust and values have flowed into the development of the model. However, from a psychological and ethical point of view, some essential aspects are still disregarded as positive or negative affection, authenticy, autonomy or resonance (Beimborn et al., 2016; Rosa, 2019). And MEESTAR is a procedural model, which does not represent its own ethical position, but only brings different ones into discussion with each other.

Beyond procedural ethics, we have to refer to models with a strong concept of acceptability (Jaensch and Nass, 2019), like for example a Kantian or a Christian perspective—equally legitimate in terms of scientific theory to start from secular or religious postulates. Therefore, we choose for the criterion of acceptability as a semantically substantial position with a transparent humanistic view of dignity. The focus lies independently of economically measurable acceptance, on the consequences for the image of man, responsibility and social coexistence. Against a legal positivist view in which laws and ethics coincide, we do not derive our ethical arguments from legal provisions, which, moreover, also vary widely internationally. It is precisely this perspective that enables a critical evaluation of rules and laws as well.

According to this view, the use of technology is acceptable if it enables every human being to live up to the responsibility given to her/him (by God or by reason) before herself/himself, before each other (and before God or before reason). The unconditional human dignity as the basis of humanity and thus of ethics is then justified, for example, in the idea of the image of God in man (Christianity), the idea of the substitution of God by man (Islam) or in the necessities of reason (Kant). Autonomy understood in this way is thus always linked to given tasks or duties, which could be justified in Kantian or religious terms (Westphal, 2016; Frick, 2019). Equal dignity belongs to every human being, but not to machines, virtual realities, cyborgs or the like. The use of technology in care must then always be a service value for the development of human beings in their individuality, sociality and triple responsibility. Such humanity should absolutely frame the logic of self-referential technology.

### Emotional Consequence and the TEO-Model (E)

Drawing on the idea of emotion-management and transformative leadership, organizational changes as AT/AR implementation in LTCFs are emotional challenges, which can trigger uncertainty, mistrust or fear within the setting. The competence of the nurse to establish a safe and healing connection with the person in need of care symbolizes a central ethical and emotional content of the care profession. On the other hand, the effects of technology use could facilitate the daily work routine/process of nurse-skilled employees and promote a pleasant organizational culture.

If certain emotions are successfully fostered (not manipulated!) by leaders in the long term, the corporate culture may also change in this direction over time and contribute to individual and organizational well-being. For leaders in the care sector it is a matter of accepting and reflecting on existing emotions within the workforce, but also of creating framework conditions that positively support the change process on an emotional level (Goleman et al., 2002; Bolton, 2005).

As described in section “Acceptance (A),” several models on technology acceptance embrace factors as attitude and behavior shaped by structural and cognitive factors. Purchasing behavior and positive emotions about AT/AR implementation are, however, susceptible to manipulation and misinformation. Concerning the legitimacy and emotional consequences for the image of caregivers as human beings, this means concretely that the use of technology is only legitimate if it does not lead to the isolation or anonymization of human contacts. If technology replaces human interaction and feelings of belonging and agency, the social nature of human beings is misled.

Collective emotions are part of the corporate culture and generally to accept. This may mean that in an organization where cautious, protective behavior has reliably led to success in the past, implementation should be undertaken in particularly slow steps, staff should be able to try out the new solution first in test settings, where mistakes do not mean serious consequences and where they have the opportunity to express concerns openly.

Furthermore, leaders—as distinct role models—should be aware of their own emotions regarding the change and reflect emotions that new tools and practices trigger in them, even before the actual implementation. However, emotions such as satisfaction or anticipation are naturally more conducive to the implementation process than fear, anger or sadness. Therefore, it is helpful to create framework conditions that promote positive emotions regarding the change of nursing practice. This can be, for example, the possibility of co-determination in the choice of technology or the co-design of the adaptation of work processes, but it can also initially mean giving space for the articulation of negative emotions on their workflow.

**FIGURE 3:**
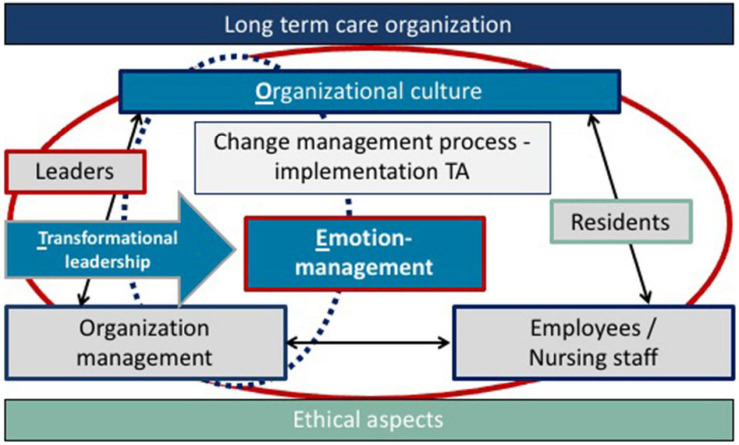
Implementation of technical assistance and transformational leadership with emotion-management in nursing practice—TEO (own elaboration).

Since AT/AR is added as a supporting factor, the former dyadic “two-way relationship” between care receiver and care giver becomes a “three-way relationship” (see Figure 3). We call this framework “TEO” for the integration of **t**ransformational leadership, **e**motion-management and **o**rganizational culture as diagnosis and iterative assessment instrument of the dimension “emotional consequence.”

In Figure 3, measures in TEO are presented that are significant for emotion-management in organizations. It should be designed like leadership around AT/AR, e.g., with regard to the question, how emotions can be created around the technology, like trust, joy, peace, harmony etc. It is also necessary, that the employees/nursing staff feel accepted and creative. The ethical aspects shed light on the iterative process of implementing a technical assistance system from the beginning. The aspects of AT/AR implementation are structured by the leader/organization management within the framework of transformational leadership and include the essential aspects of the organizational culture, management, the personnel in LTCFs and implicitly also the persons in need of care. A possible tool for implementation are workshops in which leaders and organization management specifically address how the care tasks may be changing and redistributed within the framework of change management.

To summarize, measures for an AT/AR implementation should address the following key questions:

1. What is the emotional *status quo* before running the change management process within the organization or among employees we have to consider in order to create a corresponding emotional culture?
2. What changes—especially in the caregiving relationship, communicative behavior, and health status—could each result from the use of technology?
3. To what extent may these positive/negative effects influence workflow and job satisfaction of the nursing staff as well as the organizational culture and thus promote/impair the implementation?

Under this assumption, an intended implementation would necessarily have to take into account the patient-nurse relationship, general ethical-human assessment criteria, and their interactions.

## AAE-Application to the Practical Example

Having presented our specific assessment tool of AAE, we now would like to illustrate this model along the illustrative example introduced earlier: A LTCF and the implementation of the “Care Assist Robot” (CAR).

### Acceptance (A)

According to important models of user acceptance [see section “Acceptance (A)”], this dimension represents the willingness of the employees to include CAR in their daily routines. As the TAM model and its modifications or the Almere model indicate, the acceptance depends for example on the tasks, CAR is designed for—in this context physical assistance in care work—and its perceived usefulness and how easy CAR can be operated. In this case, greater acceptance can be assumed, as CAR is supposed to reduce physical burden for caregivers and therefor performs an important care tasks that doesn’t replace social or emotional interactions between caregiver and care receiver (as, for example, it would possibly be the case with social assistive robots).

Nursing staff’s concerns about insufficient functions (like taking too much time for moving a patient) or loss of control could be addressed in terms of specific workshops for introduction and peer-to-peer-education, which relates to specific user characteristics, realistic scenarios and the social context in which the robot is used. The opportunity to try out CAR and its functions in a workshop setting (and not in urgent situations) allows employees a prevention-oriented culture in respect of shortcomings but also as arenas for experienced self-efficacy and confidence. In addition, it is important to consider the voluntariness to use CAR and if resistance (by employees but also by residents) against the robot is acceptable for leaders. Possibilities of participation in the implementation process (as mentioned in this example) and transparent information policy by the leaders are also crucial to avoid negative attitudes and perceptions of replacement.

### Ethical Acceptability (A)

Ethical acceptability in our example is more likely as CAR provides physical tasks to reduce physical impairments and mobile difficulties for the persons in need of care. However, staff members in our example are concerned about the possibility to become removed by CAR in their personal assistance.

In respect to ethics in nursing social interactions and non-maleficence of patients are roles, which cannot be compensated by a AT/AR. However, conflicts between patients and care staff in the ethical evaluation of CAR use may arise, for example, where patients prefer the use of technology to a human nursing service, while nurses see this specific service as essential to their job description and identity. Or, vice versa, a nurse may want to get rid of unpleasant care services that the patient prefers to see provided by a human. Such conflicts have consequences for organizational culture when nurses’ issues of conscience affect their motivation, job satisfaction, and identification with their work. The criterion of ethical acceptability of CAR use does not require smoothing out all such conflicts. If the use of technology with all its consequences leads to the fact that thereby the human relationship between them and patients wins, then it is acceptable. The concrete evaluation in individual cases depends on the semantics of the idea of humanity, which can be shaped differently for cultural or ideological reasons.

In our example, it is possible that patients in specific care contexts prefer the use of CAR to care by humans, for example out of shame, and therefore regard this arrangement as morally preferable. For the self-image of nursing staff, the use of CAR can then also be seen as a facilitation, because encounters with shame can be avoided. Such a substitution of human care by robotics is acceptable in principle, if it is always clear that an ethical evaluation on the use of CAR does not itself attribute a moral quality to this concrete robotics. The use may be acceptable, but the robotics itself is never morally good or bad. Other positions are conceivable here, for example in animism (Kaplan, 2004; Hornyak, 2016).

Who is CAR in comparison to the nurse and other employees in terms of human image and nursing ethics? How is responsibility in care tasks attributed between staff and CAR? Once such questions have been clarified in the organization (clear limits have been set for the use of robots; there is no replacement of human communication; machines are not colleagues and questions of liability have been clarified), the use of CAR could be ethically supported in principle under these conditions if it actually relieves the workload of the scarce nursing staff and if this robotics is easy to operate.

### Emotional Consequences (E)

In our illustrative example employees reported various emotions with regard to CAR: anxiety, anger, confusion but also curiosity. While some of them are frustrated and disappointed, because the management is buying robots instead of creating new jobs, others are worried about being replaced by a CAR with the possible loss of their job. Possible unexpected reactions could arise on the part by generating even more negative emotions among the staff, developing a disturbed trust relationship with the management, leading to for example increased sick days or staff turnover.

The application of TEO (including transformative leadership, emotion-management and organizational culture) in its practical feasibility means that leadership is associated with an emotional reaction of the employees and has corresponding emotional effects that have presumably hardly been perceived in leadership management.

In the illustrative example of a LTCF, knowing exactly what contributes to a professional workflow and satisfaction of care staff in their job, are significant steps to move together positively in the direction of change management (e.g., implementation of AT/AR). In the case of the existing negative emotions with regard to CAR, it is important that the manager promotes positive emotions and has a calming effect with less intensity, satisfaction and serenity and that their role model function again exemplifies trust through authentic, honest and beneficial communication processes as well as the emphasis on joint positive performances, so that a change from negative can be converted into positive emotions.

With the focus particularly on *emotion-management*, timing and emotions must be thought together. The change management process in our example starts a long time before the implementation process of the AT/AR (e.g., planning budget for CAR, negotiation with the manufacturers). In workshops (as already mentioned with regard to *Acceptance*) the transformational leadership has to create an emotional vision of CAR implementation. Caregivers want to be informed and active part of the process and transparent, authentic and communicative information policy by the leading management. This means to create emotions around the robot, like trust, joy, peace, harmony etc., were caregivers feel accepted, creative and are able to flourish.

The application of our three-stage system for the introduction of the Care Assist Robot has shown that acceptance, ethical acceptability and emotions each make criteria for an implementation transparent, which allow a holistic evaluation also in a human perspective. These critical criteria can now be easily merged. They are the compass for the management of LTCF to design a responsible transformational change process in response to AT/AR implementations.

## Discussion and Outlook

In the future, residential care will increasingly face the challenge of successfully implementing digital technologies. This can especially be expected for LTCFs, as multiple assistive technologies and robots promise new possibilities for maintaining the quality of life of vulnerable residents as well as facilitation for professional caregivers in their daily work. Several studies underlined the importance of different acceptance dimensions in the care sector and specific outcomes for different user groups.

From our perspective, successful implementation by organizational leaders also has to take into account the existing organizational culture and to support employees in these changes beyond traditional concepts of technology acceptance, especially on an emotional level and in respect of ethical values in nursing. Compared to other industries, the implementation of AT/AR in LTCFs has to consider specific emotional conditions in care settings in terms of vulnerable residents with daily care needs, shortage of professional caregivers and ethical social rules in nursing. In the context of facilitators and barriers contributing to the AT/AR deployment, the stakeholder-entities are decisive too: The aim of AT/AR implementation is frequently to evoke positive emotionality in patients and employees and thus to support the health balance of older or cognitively impaired people. Ideally, these generated effects would have an equally positive effect on professional caregivers and, in a broader sense, the entire organization, including the prevailing work culture.

Thus, we argue here for a more ethical and emotion-led leadership and management strategies in care institutions to enable modern organizations to adopt a constructive and reflexive attitude toward technology without, however, being manipulative. We offer a humanistic compass with the evaluation criterion of AAE (acceptance, ethical acceptability and emotional consequences), which includes the idea of humanity and social coexistence for the solution of concrete individual questions in care practice. In doing so, we avoid a paternalistic narrowing, as local norms and organizational cultures should be considered.

To avoid unpredictable/unintended results and resistance, employees should be stimulated by change and involved in a participative way. In this respect, an intentional utilization of factors which foster facilitator-categories is very beneficial for the entire institution. While the concept of “emotion-management” explores human emotions at the workplace and conceptualize a new management approach (Bolton, 2005), “transformational leadership” underlines the role model function of leaders, attractive goals and motivation, or stimulation for employees (Bass and Avolio, 1994). We have translated the emotional consequences in the AAE approach into a three-perspective heuristic model “TEO” that integrates previous prominent approaches on transformative leadership, prescriptive emotion-management and organizational culture on the issue of technology implementation. Emotional consequences captured by the perspective of TEO can potentially support organizations in developing appropriate implementation guidelines and provide ideas for a common value discussion. In addition, adapted to the respective institutional framework conditions, it can represent an initial diagnostic or rather iterative assessment blueprint for understanding and improving change management during the hole implementation process. For example, ethical and emotional-based questions could be included in internal surveys and emotional resources could be considered in the evaluation.

Our findings should be interpreted while considering certain limitations. We are aware that our AAE-model is initially a working hypothesis that deserves further development. Thus, a fourth or fifth essential perspective could be added to AAE as other acceptance logics could be applied. The ethical acceptability model could be given a different semantics than ours (humanistic-Christian-Kantian), i.e., utilitarian, anthroposophical, etc. In addition, the specific organizational context (funding principles, ethical codex, number and skills of employees and clients, number of residents with cognitive decline) plays an important role for the debate around the priority of robots vs. human care providers. In addition, leadership styles like transformational leadership can be taught to leaders by individual coaching or peer counseling, but it needs to be practiced and internalized, which takes time and support. Another limitation lies in the implementation of the model, which first of all means an additional effort (time, costs, intensity) for the management.

In addition, there is still a need for research regarding the question which contextual conditions in the care sector shape a resonant relationship between leading attitudes and behavior by the management and emotions by employees (Plummer, 2018; Rosa, 2019). A deeper insight here could explore, which emotions are particularly helpful and which ones hinder technology implementation. Here, comprehensive empirical analyses of successful and unsuccessful implementation attempts in care organizations are recommended.

## Author Contributions

AF prepared the general concept and outline of the publication, the introduction, the theoretical part on relevant concepts of technology acceptance as well as discussion and conclusions. EN, A-KP, AZ, and CH prepared the whole theoretical part on emotion-management, change management and role of leadership. All authors made comments, suggestions and corrections to the rest of the article.

## Conflict of Interest

AZ was employed by the company Youse GmbH. The remaining authors declare that the research was conducted in the absence of any commercial or financial relationships that could be construed as a potential conflict of interest.

## Publisher’s Note

All claims expressed in this article are solely those of the authors and do not necessarily represent those of their affiliated organizations, or those of the publisher, the editors and the reviewers. Any product that may be evaluated in this article, or claim that may be made by its manufacturer, is not guaranteed or endorsed by the publisher.

## Footnotes

1 Defined as a robot “that performs useful tasks for humans or equipment excluding industrial automation applications.” Service robots can be categorized as medical robots, logistic robots or field robots (ibid). Further categorizations of robot-based services differ between (1) assistive robots primarily used for physical applications, (2) socio-emotional robots for companionship and (3) robots for health and safety monitoring (Sharkey and Sharkey, 2012; Prabuwono et al., 2017; Meyer et al., 2020).

2 Defined as “a facility that provides room and board, as well as management of chronic medical conditions and 24-h assistance with ADLs in patients who are physically and/or cognitively impaired” (Klein et al., 2013; Sanford et al., 2015: p. 182).
